# Adjunctive topical hemostatic agents combined with renorrhaphy versus renorrhaphy alone during partial nephrectomy: a systematic review and meta-analysis

**DOI:** 10.1007/s00345-026-06679-5

**Published:** 2026-08-21

**Authors:** Jose Arnaldo Shiomi da Cruz, Artur De Souza Almeida, Bruno Damico Terada, Felipe Giraldo Alvarez Gonçalves, Breno Cordeiro Porto, Rodrigo Afonso Da Silva Sardenberg, Tiago Aparecido Silva, Ronaldo Soares Maia, Fernando Gonçalves de Almeida, Murilo de Almeida Luz

**Affiliations:** 1 Department of Urology, Federal University of São Paulo Medical School (UNIFESP/EPM), Botucatu St., 740 - Room 504, Sao Paulo, 04023-900 Brazil; 2 Department of Urology, Ninth of July University, Sao Bernardo Do Campo, Brazil; 3 Research Institute - Hapvida NotreDame, Sao Paulo, Brazil; 4 Department of Urology, Icahn School of Medicine at Mount Sinai, New York, NY USA

**Keywords:** Partial nephrectomy, Hemostatic agents, Systematic review, Meta-analysis

## Abstract

**Purpose:**

Partial nephrectomy (PN) is the standard of care for T1 renal tumors, offering oncological outcomes equivalent to radical nephrectomy while preserving renal function. Intraoperative hemostasis remains a critical challenge, as warm ischemia time is independently associated with postoperative renal functional decline. Topical hemostatic agents have been increasingly adopted as adjuncts to renorrhaphy; however, their incremental benefit over suture alone remains unestablished. This study aimed to compare the efficacy and safety of hemostatic agents combined with renorrhaphy versus renorrhaphy alone in patients undergoing PN.

**Materials and methods:**

This systematic review and meta-analysis followed PRISMA guidelines and the Cochrane Handbook, with prospective registration in PROSPERO (CRD420261396802). MEDLINE, Embase, CENTRAL, Web of Science, and Scopus were searched from inception to March 15, 2026, for RCTs and comparative observational studies comparing hemostatic agents combined with renorrhaphy versus renorrhaphy alone in adult PN patients. The primary outcome was blood transfusion rate (BTR); secondary outcomes included estimated blood loss (EBL), hemorrhagic complications (HC), and urinary leakage (UL). A random-effects model with REML estimation was used. Risk of bias was assessed with RoB 2 and ROBINS-I V2; certainty of evidence was graded using GRADE.

**Results:**

Fifteen studies (2 RCTs, 13 observational) comprising 3,408 patients were included from 2,281 identified (1,265 screened after duplicate removal). No significant difference was observed for BTR (RR 0.96; 95% CI 0.59–1.57; I^2^ = 44.7%; 95% prediction interval 0.24–3.90), HC (RR 0.73; 95% CI 0.40–1.35; I^2^ = 47.6%; PI 0.14–3.83), or UL (RR 1.36; 95% CI 0.56–3.28; I^2^ = 0.0%; PI 0.45–4.09). EBL heterogeneity was considerable (I^2^ = 97.8%) and irreducible on leave-one-out (lowest I^2^ = 84.1%); it is therefore reported narratively, with study-level differences ranging from − 120 to + 57 mL. No publication bias was detected (Egger p = 0.598).

**Conclusions:**

Current evidence does not demonstrate a benefit of adjunctive hemostatic agents over renorrhaphy alone. With wide intervals and low certainty for most outcomes, these data reflect an absence of evidence of benefit rather than evidence of absence, and a clinically relevant effect cannot be excluded. Routine use is not supported; adequately powered trials stratified by tumor complexity and agent class are warranted.

**Supplementary Information:**

The online version contains supplementary material available at 10.1007/s00345-026-06679-5.

## Introduction

Renal cell carcinoma (RCC) represents the most common malignancy of the kidney, accounting for approximately 434,840 incident cases worldwide in 2022, and ranking as the sixth most common cancer in men and the ninth in women in the United States [1]. Its incidence has risen steadily at approximately 1% per year, a trend largely attributable to the widespread adoption of cross-sectional abdominal imaging and the consequent increase in incidental detection of small renal masses, now documented in 37–61% of newly diagnosed patients [1, 2].

Partial nephrectomy (PN) has emerged as the standard of care for clinical T1 renal tumors and selected T2 lesions, demonstrating oncological outcomes equivalent to radical nephrectomy while preserving long-term renal function and reducing the risk of chronic kidney disease, cardiovascular morbidity, and overall mortality [3].

Despite its established benefits, PN remains technically demanding, and intraoperative hemostasis represents one of its most critical challenges. Tumor resection is typically performed under hilar clamping, exposing the kidney to warm ischemia, the duration of which is independently associated with postoperative renal functional decline [4, 5]—though emerging evidence suggests that preserved parenchymal volume and pre-existing renal comorbidities may be equally or more relevant determinants of long-term functional outcomes [6–8].

Renorrhaphy—the multilayer suture repair of the resection bed—constitutes the cornerstone of parenchymal hemostasis and collecting system closure. In an effort to further optimize hemostasis, topical agents—including fibrin sealants, oxidized cellulose matrices, and gelatin-thrombin composites such as TachoSil**®** and Floseal**®**—have been increasingly adopted as adjuncts to renorrhaphy [9, 10]. The role of these agents may be further amplified by the progressive expansion of PN indications to anatomically complex tumors and by the emerging practice of omitting the parenchymal closure layer in select exophytic cases, where topical agents would bear greater hemostatic responsibility [11, 12]. Nevertheless, their routine use entails considerable added costs and potential risks, including local inflammatory reactions and foreign body responses, without a clearly established clinical benefit over suture alone [13–16].

The existing literature remains heterogeneous and largely composed of retrospective, single-center studies, precluding definitive conclusions regarding the incremental value of these agents. Therefore, the present systematic review and meta-analysis aims to evaluate and compare the efficacy and safety of hemostatic agents combined with renorrhaphy versus renorrhaphy alone in patients undergoing PN, with emphasis on blood transfusion rate (BTR), estimated blood loss (EBL), hemorrhagic complications (HC), and urinary leakage (UL).

## Materials and methods

### Search strategy

This systematic review and meta-analysis was conducted and reported in accordance with the Cochrane Handbook for Systematic Reviews of Interventions [17] and the PRISMA guidelines [18], and was prospectively registered in PROSPERO (CRD420261396802). A comprehensive literature search was performed across MEDLINE (via PubMed), Embase, CENTRAL, Web of Science, and Scopus from database inception to March 15, 2026 (date of the final search), designed to identify studies comparing hemostatic agents combined with renorrhaphy versus renorrhaphy alone in PN. No language or publication-date restrictions were applied. The full search strategy is detailed in eTable 1. Reference lists of all included studies were additionally screened to identify any eligible trials not captured by the electronic search.

### Eligibility criteria

Studies were eligible for inclusion if they: (1) involved adult human participants undergoing PN; (2) were designed as RCTs or observational studies with a comparative design; and (3) directly compared hemostatic agents combined with conventional renorrhaphy versus renorrhaphy alone. Studies were excluded if they: (1) involved non-human subjects; (2) did not report any prespecified outcome of interest; (3) were case reports, case series, editorials, or non-comparative designs; (4) compared hemostatic agents alone versus suture without a combined-modality arm; or (5) did not report outcomes separately for each group.

### Outcomes

The primary outcome was BTR, defined by each study's own criteria, as no universal transfusion threshold was applied. Because transfusion triggers vary between institutions and have become more restrictive over the two decades spanned by the included studies, the endpoint reflects both physiological bleeding and local transfusion policy—a limitation addressed in the Discussion.

Secondary outcomes included HC, EBL, and UL. HC encompassed any hemorrhagic complication requiring intervention, including postoperative bleeding requiring transfusion, angiographic embolization for pseudoaneurysm or arteriovenous fistula, and reintervention for hemorrhage. EBL was extracted as reported by each author, and UL was defined as any postoperative urinary extravasation or fistula requiring clinical attention Table 1.

**Table 1:**
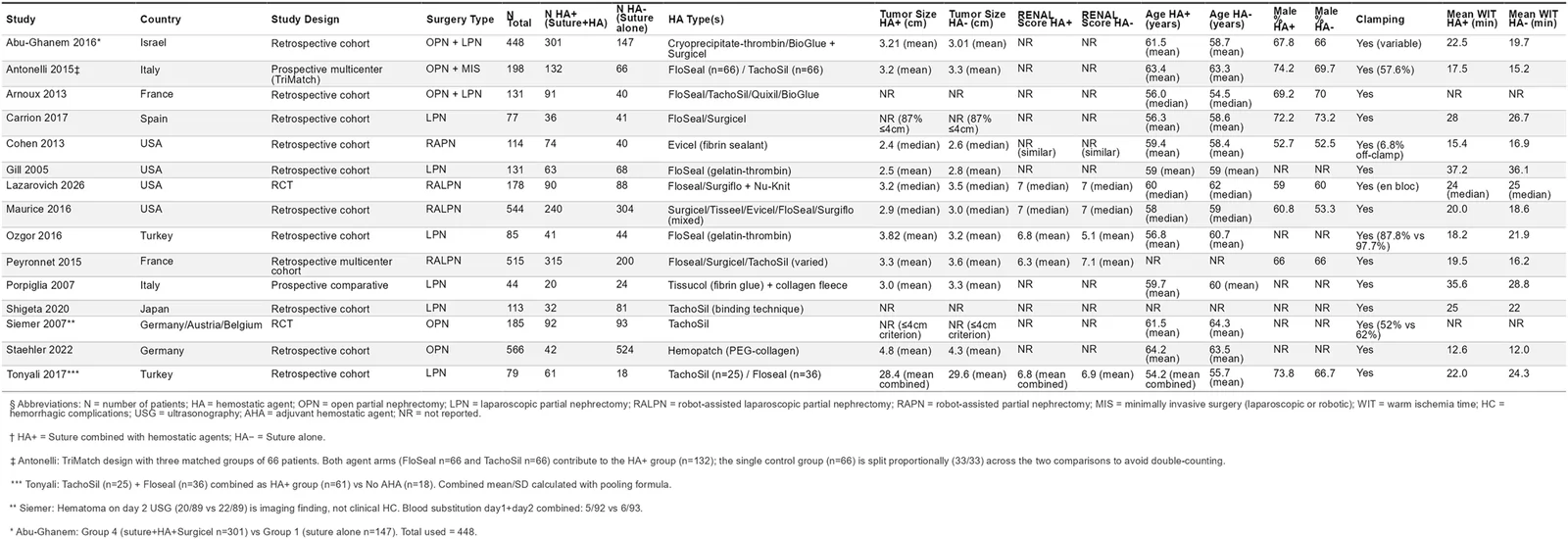
Characteristics of studies included in the meta-analysis

### Study selection

After deduplication using EndNote Online 20 (Clarivate Analytics, Philadelphia, PA, USA) [19], two independent reviewers screened titles and abstracts, with discrepancies adjudicated by a third reviewer. Potentially eligible studies proceeded to full-text review by the same two reviewers, with disagreements resolved by consensus or third-party adjudication. No artificial intelligence or automation tools were employed at any stage Table 2.

**Table 2:**
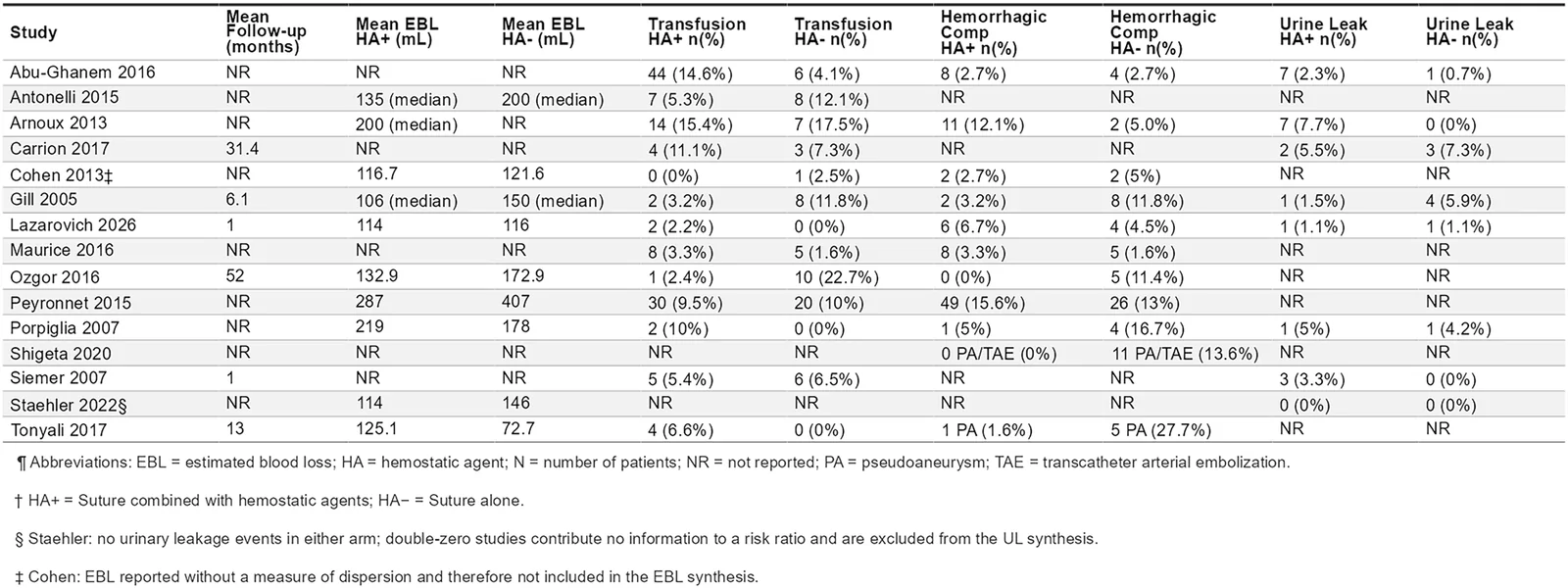
Outcomes of studies in the meta-analysis

### Data extraction and quality assessment

Two reviewers independently extracted data using a standardized, predefined form, with discrepancies resolved by a third reviewer. Extracted variables included study design, sample size, patient demographics, surgical approach, type of hemostatic agent, and all prespecified outcomes. Risk of bias was assessed independently by two reviewers using RoB 2 [20] for RCTs and ROBINS-I V2 [21] for observational studies, with disagreements resolved by structured consensus. The overall certainty of evidence was evaluated using the GRADE framework [22].

### Statistical analysis

Dichotomous outcomes were expressed as risk ratios (RR) and continuous outcomes as mean differences (MD), both with 95% CIs, using a random-effects model (REML) selected a priori. Heterogeneity was appraised without a fixed cut-off, as the Cochrane Handbook cautions that I^2^ thresholds can mislead and that τ^2^ has no established reference value [17]. We interpreted I^2^ using the Handbook's graded ranges and its 95% confidence interval, derived by the test-based method of Higgins and Thompson, alongside the magnitude and direction of effects; reported τ^2^ as the between-study variance on the outcome scale; used Cochran's Q only as an index of evidence for, not magnitude of, heterogeneity; and calculated 95% prediction intervals for outcomes with ≥ 5 studies to convey the dispersion of underlying effects. Where heterogeneity was considerable and persisted across pre-specified leave-one-out and Baujat analyses, we reported the outcome narratively rather than as a pooled estimate. Subgroup differences were tested formally under the random-effects model.

Randomized and non-randomized studies were pooled in a single random-effects model, which assumes a distribution of underlying effects rather than a common one and therefore accommodates design-related variation. To keep this transparent, study design was pre-specified as a subgroup variable so that design-specific estimates and any subgroup difference could be inspected; non-randomized studies were appraised with ROBINS-I V2 on the same effect scale as the randomized evidence; and certainty of evidence was graded separately by design under GRADE.

Random-effects meta-regression (REML) was planned for two prespecified moderators—mean tumor size and mean RENAL score [23]. As the Handbook advises against meta-regression with fewer than ten studies and recommends ~ ten studies per covariate, and no model met this threshold, these analyses are exploratory and hypothesis-generating only, reported in the Supplement and used for no inferential claim [17]. Subgroup analysis by agent class was planned but proved unfeasible, as several studies pooled multiple agents or did not specify the product (eTable 3). Models were fitted where a moderator was reported by ≥ 3 studies, with R^2^ and residual I^2^ reported.

For studies reporting multiple intervention arms against a single control group, the control group sample size was proportionally divided among intervention arms to avoid unit-of-analysis errors and double-counting of participants, in accordance with guidance from the Cochrane Handbook [17].All analyses were performed using R (version 4.0.0; R Core Team, 2020) [24] with RStudio (version 1.4.1106; RStudio Team, 2021) [25].

## Results

### Study selection and characteristics

**Fig. 1:**
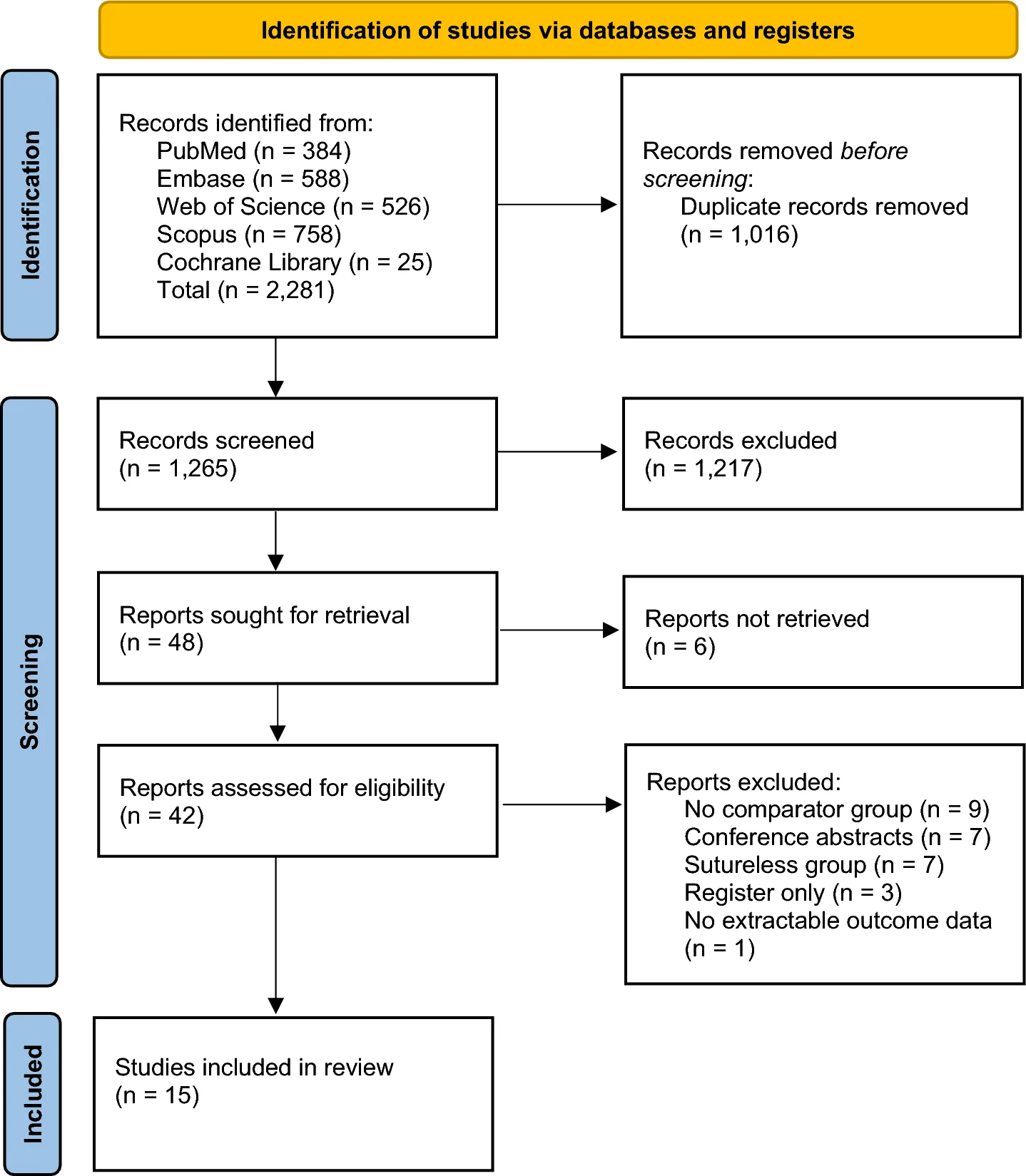
PRISMA 2020 flow diagram for new systematic reviews inclused searches of databases and registers only

The included studies comprised two RCTs and thirteen comparative observational studies (2005–2026), spanning open, laparoscopic, and robot-assisted approaches (Fig. 1). Agents included gelatin–thrombin matrices (FloSeal®), fibrin-coated collagen patches (TachoSil®), polyethylene-glycol-coated patches (Hemopatch®), fibrin sealants, and oxidized cellulose/collagen fleece; several studies pooled agents within one arm or did not specify the product, precluding subgroup analysis by class (eTable 3). In total, 1,630 participants received hemostatic agents plus renorrhaphy and 1,778 renorrhaphy alone. A completed PRISMA 2020 checklist is provided in eTable 2.

### Meta-analysis

#### Primary endpoint

**Fig. 2:**
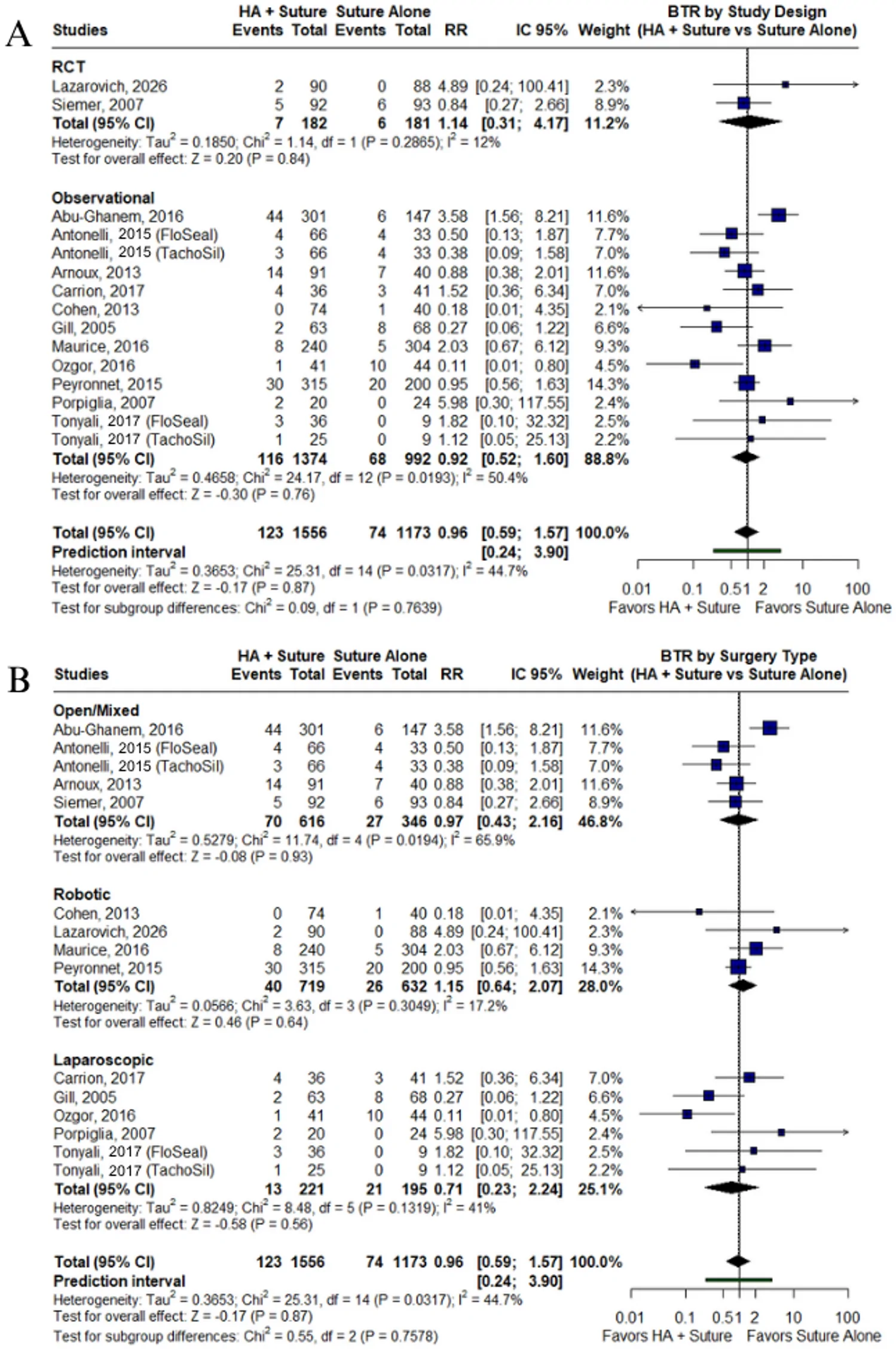
Forest plots for blood transfusion rate (BTR) comparing hemostatic agents (HA) plus suture versus suture alone, stratified by (**A**) study design (RCT vs. observational) and (**B**) surgical approach (open/mixed, robotic, laparoscopic). Random-effects model; RR = risk ratio; CI = confidence interval. Overall pooled RR 0.96 (95% CI 0.59–1.57; I² = 44.7%; 95% prediction interval 0.24–3.90)

When comparing BTR between groups, no significant difference was observed (RR 0.96; 95% CI 0.59–1.57; p = 0.87; I^2^ = 44.7%, 95% CI 0.0–69.8%; Q = 25.31, df = 14, p = 0.032) (Fig. 2). The 95% prediction interval (0.24–3.90) and the confidence interval both remain compatible with a clinically useful reduction in transfusion as well as with an increase; a benefit is therefore not excluded.

Subgroup analysis by study design showed no significant effect in either the randomized (k = 2; RR 1.14; 95% CI 0.31–4.17; I^2^ = 12%) or observational subgroup (k = 13; RR 0.92; 95% CI 0.52–1.60; I^2^ = 50.4%, 95% CI 6.2–73.7%), with no subgroup difference (p = 0.764). The randomized subgroup, however, rests on two trials contributing thirteen events and is uninformative in isolation; the absent subgroup difference reflects the width of both intervals rather than concordance. Analyses by surgical approach were likewise non-significant across open/mixed (RR 0.97; 95% CI 0.43–2.16; I^2^ = 65.9%), robot-assisted (RR 1.15; 95% CI 0.64–2.07; I^2^ = 17.2%), and laparoscopic procedures (RR 0.71; 95% CI 0.23–2.24; I^2^ = 41.0%; subgroup difference p = 0.758).

Given the substantial heterogeneity observed, pre-specified sensitivity analyses were performed. In the LOO analysis, omission of Abu-Ghanem (2016) [10] yielded the greatest reduction in heterogeneity (I^2^ = 16.0%), with a modest shift in the pooled estimate (RR 0.86; 95% CI 0.62–1.19). Sequential exclusion of all remaining studies maintained I^2^ between 35.9% and 48.6%, with no reversal of effect direction or emergence of statistical significance, indicating the result is not driven by any single trial. The Baujat plot corroborated these findings, identifying Abu-Ghanem (2016) [10] as the primary outlier in both heterogeneity contribution and influence on the pooled estimate, while the remaining studies clustered in the lower-left quadrant (eFigures 1–2).

**Fig. 3:**
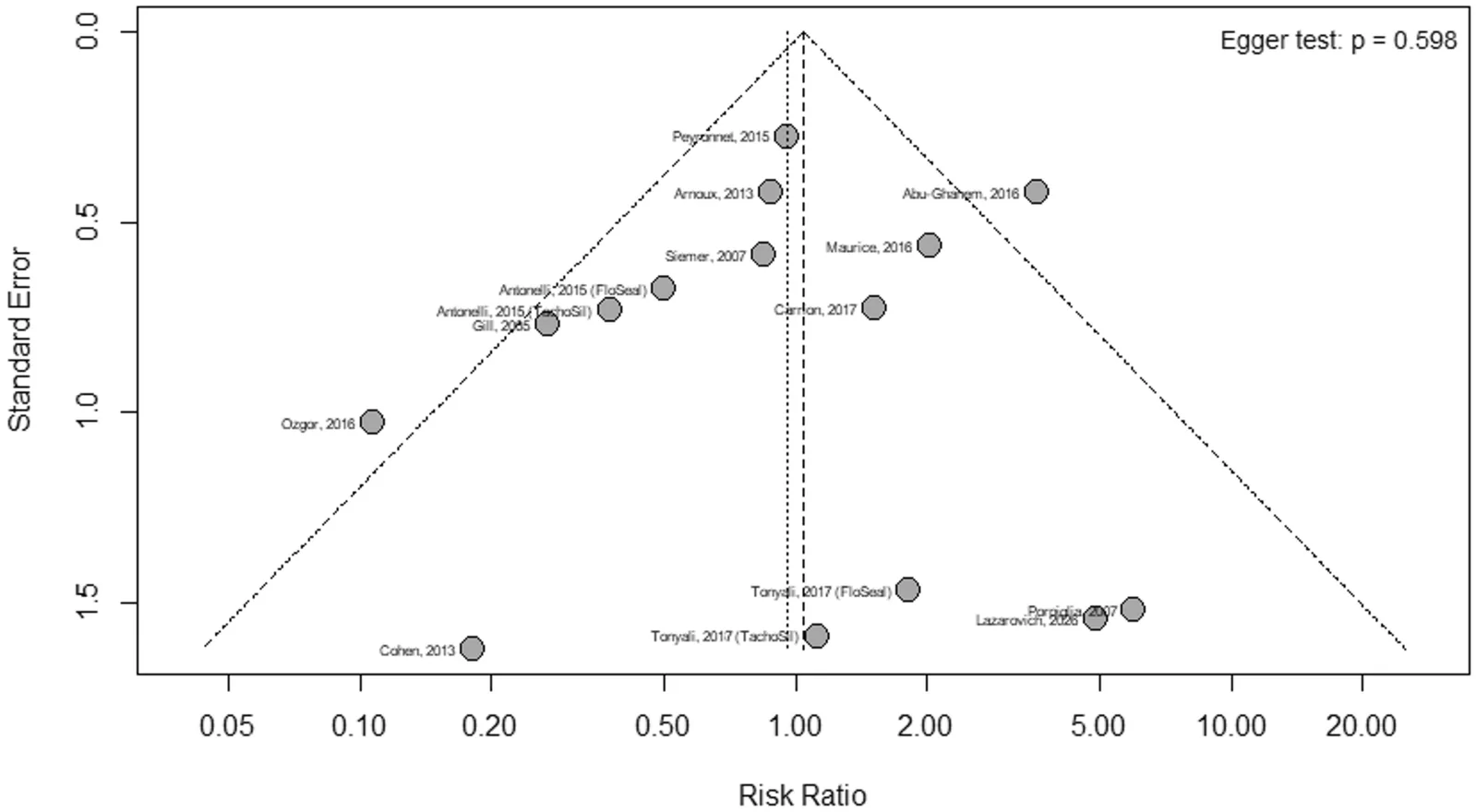
Funnel plot for assessment of publication bias in the blood transfusion rate outcome. Egger's test p = 0.598, indicating no evidence of significant publication bias or small-study effects

Publication bias was assessed through funnel plot inspection and the Egger test (p = 0.598), providing no evidence of selective reporting. Visual inspection revealed an approximately symmetric distribution of studies within the funnel (Fig. 3).

### Secondary outcomes

**Fig. 4:**
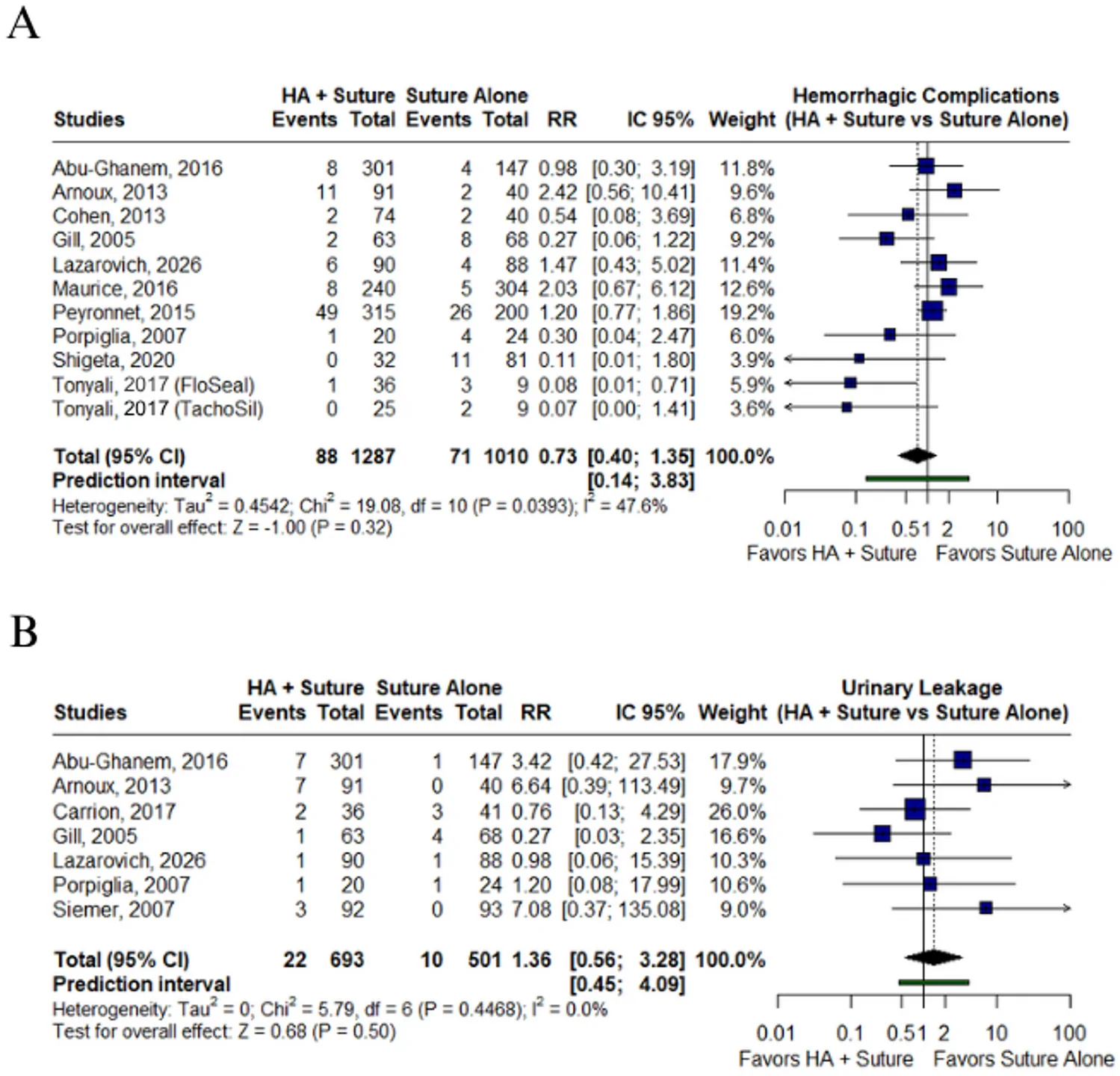
Forest plots for (**A**) hemorrhagic complications (HC) and (**B**) urinary leakage (UL) comparing hemostatic agents (HA) plus suture versus suture alone. Random-effects model; RR = risk ratio; CI = confidence interval. HC: RR 0.73 (95% CI 0.40–1.35; I² = 47.6%). UL: RR 1.36 (95% CI 0.56–3.28; I² = 0.0%)

No significant difference was observed for HC (k = 10 studies, 11 comparisons; RR 0.73; 95% CI 0.40–1.35; I^2^ = 47.6%, 95% CI 0.0–73.9%; PI 0.14–3.83) or UL (k = 7; RR 1.36; 95% CI 0.56–3.28; I^2^ = 0.0%, 95% CI 0.0–70.8%; PI 0.45–4.09) (Fig. 4); both prediction intervals span substantial benefit and harm.

For EBL, heterogeneity was considerable and not attributable to any single study (k = 8; I^2^ = 97.8%, 95% CI 96.9–98.4%; τ^2^ = 3,385.80), remaining ≥ 84% across all leave-one-out iterations. Study-level differences ranged from − 120.30 mL (Peyronnet 2015) [26] to + 57 mL (Tonyali 2017) [27], with a 95% prediction interval of − 162.11 to + 131.56 mL. Following Handbook guidance that an average effect may mislead when variation is considerable [17], we do not report a pooled mean difference as a principal finding; the exploratory estimate (MD − 15.33 mL; 95% CI − 57.94 to 27.28) is shown in eFigure 3.

Given the considerable heterogeneity, LOO and Baujat analyses were performed (eFigures 4, 5). Omitting Peyronnet (2015) [26], which carried 84.6% of the common-effect weight, produced the largest reduction, yet I^2^ remained at 84.1%—still considerable. Heterogeneity was therefore neither driven by nor resolvable through any single study, the principal reason a pooled estimate is not advanced.

For HC, omission of Tonyali (2017) [27] produced the greatest heterogeneity reduction (I^2^ = 34.7%), while omission of Peyronnet (2015) [26] yielded the greatest shift in the effect estimate (RR 0.62), reinforcing a potential protective trend that nonetheless did not reach statistical significance. The result remained non-significant across all LOO iterations. The Baujat plot identified Tonyali (2017) [27] as the primary contributor to heterogeneity and Peyronnet (2015) [26] as the greatest influence on the pooled estimate, with Maurice (2016) [28] and Gill (2005) [29] showing moderate contributions and the majority of studies clustered near the origin (eFigures 6, 7).

### Meta-regression

Random-effects meta-regression was performed to assess tumor size and RENAL score as potential moderators of the bleeding-related outcomes for which data were available (eFigures 8–13). For tumor size, no significant association was identified with BTR (k = 10; OR per + 1 cm = 1.30; 95% CI 0.06–27.42; p = 0.868; R^2^ = 0%; residual I^2^ = 69.2%), HC (k = 7; OR per + 1 cm = 2.59; 95% CI 0.63–10.75; p = 0.189; R^2^ = 0%; residual I^2^ = 2.9%), or EBL (k = 6; MD slope per + 1 cm = − 20.1 mL; 95% CI − 83.4 to 43.2; p = 0.534; R^2^ = 0%; residual I^2^ = 87.4%).

For RENAL score, a positive association with BTR was estimated (k = 5; OR per + 1 point = 23.66; 95% CI 2.62–213.97; R^2^ = 100%). This is not interpretable as a dose–response relationship: fitted on five studies for one covariate, its implausible estimate, two-order-of-magnitude interval, and R^2^ of 100% are characteristic of overfitting, and neither its significance nor its apparent explanation of heterogeneity should be read literally. No association was found for HC (k = 3; p = 0.457) or EBL (k = 3; p = 0.273). All models (eFigures 8–13) hypothesis-generating only.

### Risk of bias assessment

**Fig. 5:**
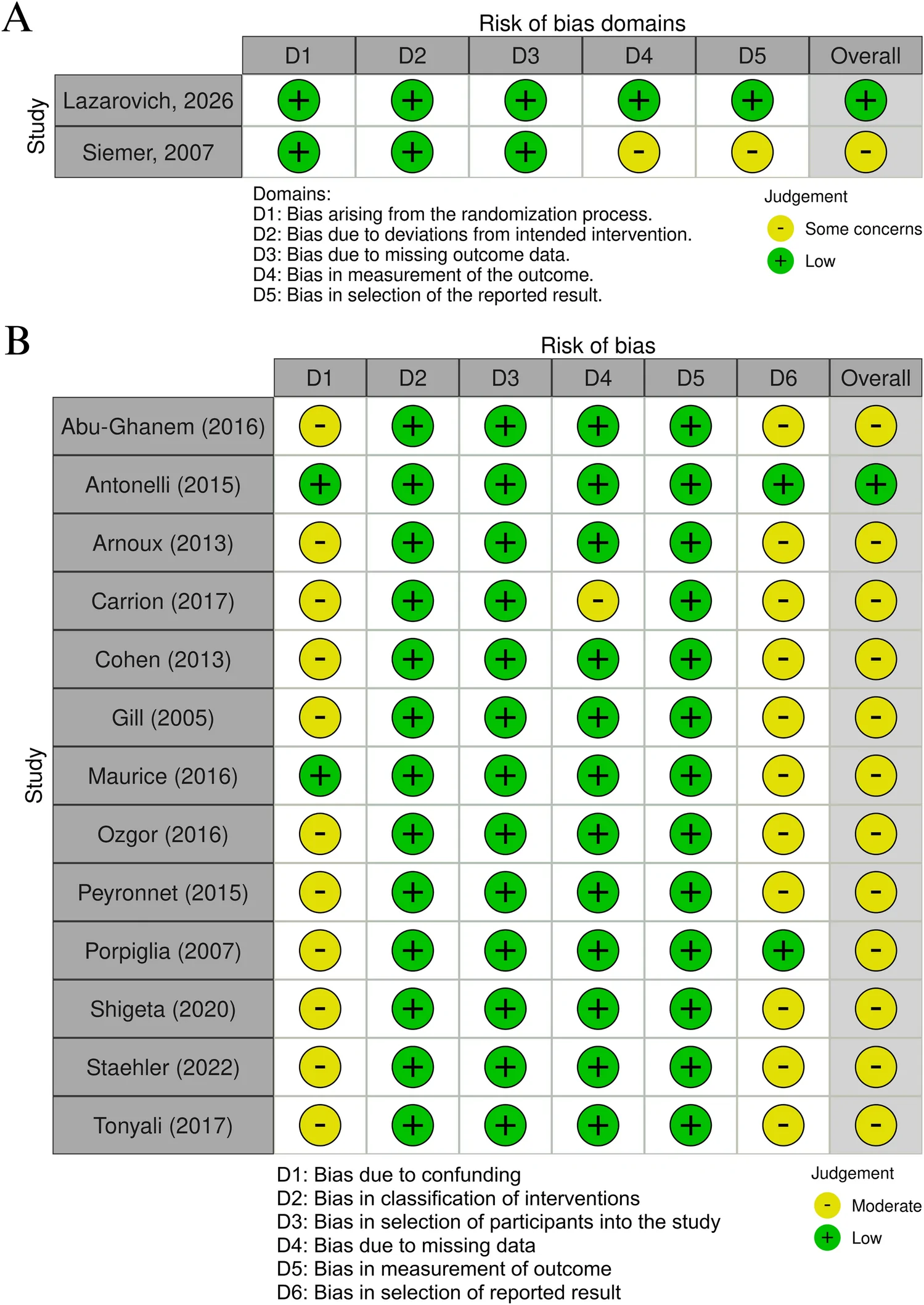
Risk of bias assessment of included studies. (**A**) RoB 2 tool for the two randomized controlled trials across five domains (D1–D5: randomization process, deviations from intended intervention, missing outcome data, measurement of the outcome, selection of the reported result) with overall judgment. (**B**) ROBINS-I V2 tool for the thirteen observational studies across six domains (D1–D6: confounding, classification of interventions, selection of participants into the study, missing data, measurement of outcome, selection of the reported result) with overall judgment

The two RCTs were assessed using the RoB 2 tool [20]. Lazarovich (2026) [30] presented a low overall risk of bias across all domains. Siemer (2007) [31] raised some concerns, particularly regarding outcome measurement (D4) and selection of the reported result (D5). The thirteen observational studies were assessed using the ROBINS-I V2 tool[21]. Antonelli (2015) [9] presented a low overall risk of bias, while the remaining twelve studies [10, 26–29, 32–38] presented moderate overall risk, predominantly driven by concerns in confounding (D1) and selection of the reported result (D6) (Fig. 5).

### GRADE assessment

Certainty was moderate for BTR in randomized trials, very low for BTR in non-randomized studies, and low for HC, EBL, and UL. Downgrading reflected risk of bias in observational studies (chiefly confounding and selective reporting), inconsistency (EBL), and imprecision, with every interval crossing the null. The certainty is thus insufficient to exclude a clinically relevant effect on any outcome. The full profile is provided in the Supplement.

## Discussion

Partial nephrectomy has established itself as the preferred surgical approach for localized renal tumors, offering oncological outcomes equivalent to radical nephrectomy while preserving nephron mass and long-term renal function [1–3, 39]. Intraoperative hemostasis represents one of its most critical technical challenges, with direct consequences on postoperative renal functional outcomes [4, 40, 41], further compounded by the transition to minimally invasive platforms, where reduced tactile feedback and increased technical complexity render parenchymal hemostasis more demanding [15, 42]. Topical hemostatic agents were incorporated into urological practice based on early promising small-scale studies and the rationale that supplementing renorrhaphy with an additional hemostatic layer could optimize perioperative outcomes, though this adoption occurred without the methodological rigor applied to other surgical innovations [10, 30, 43, 44]. The costs associated with these agents range from hundreds to thousands of dollars per procedure, with no cost-effectiveness data demonstrating benefit over renorrhaphy alone, and their use carries potential risks including foreign body reactions, granuloma formation, fibrosis, ureteral obstruction, and the potential to serve as an infectious nidus when incompletely absorbed [13–16].

Our primary endpoint was perioperative BTR, the most clinically relevant and objective measure of hemostatic efficacy. The pooled analysis demonstrated no statistically significant difference between groups (I^2^ = 44.7%). This finding is physiologically consistent with the mechanism of renorrhaphy itself: multilayer suture closure directly addresses the primary bleeding sources—interlobar vessels and segmental arterial branches—through mechanical compression and vascular ligation, whereas topical agents act exclusively at the parenchymal surface level [10, 45, 46]. Contemporary BTR in high-volume centers range between 2–10%, a dramatic reduction from the 15–20% rates of early open series attributable to surgical technique refinement rather than hemostatic adjuvants [3, 47–49]. When baseline transfusion rates are already in single digits, detecting a small relative effect would require samples far larger than those available. Our interval nonetheless remains compatible with a clinically useful reduction: the evidence is insufficient to demonstrate benefit, not sufficient to exclude it.

The concordance of subgroup analyses should not be over-read as mutual corroboration. The randomized subgroup rests on two small trials whose interval (RR 1.14; 95% CI 0.31–4.17) spans substantial benefit and harm, and cannot confirm or refute the observational signal. The observational estimate, in turn, is vulnerable to confounding by indication: agents are preferentially used in the more demanding cases—friable parenchyma, hilar or deeper resections, anticoagulation—so intervention arms are enriched for higher bleeding risk, biasing the association toward the null and potentially masking a genuine benefit. No included study adjusted for indication, and the predominance of confounding concerns on ROBINS-I V2 reflects this. We therefore treat this as a live alternative explanation for the null rather than a residual caveat.

For EBL, heterogeneity was considerable and irreducible (τ^2^ = 3,385.80; prediction interval − 162 to + 132 mL), and we decline to advance a pooled estimate that a − 15 mL point value cannot meaningfully summarise. The variability is itself informative, exposing a limitation of the literature rather than of the analysis: EBL is notoriously subjective, differing 30–50% from gravimetric methods [50–53], and is strongly shaped by tumor complexity, which most studies neither reported nor adjusted for [54–57]. As reported across this literature, EBL is not measured on a scale reliable enough to support pooling, and no conclusion about blood loss—in either direction—can be drawn from it.

For HC, although a numerical trend toward a protective effect was observed, this did not reach statistical significance across any LOO iteration. Late HC following PN arise from mechanisms inaccessible to topical agents: tumor size and complexity, not hemostatic technique, are the primary risk factors, with larger tumors requiring deeper suture placement into vascular structures that surface-applied agents cannot reach [58–60]. Consistent with this, the exploratory meta-regressions for HC were uninformative and, given the few contributing studies, are not used to support any claim.

Urinary leakage showed no significant difference between groups, with complete absence of heterogeneity. This result is physiologically intuitive: UL results from inadequate closure of the collecting system, whose prevention depends entirely on meticulous deep suture of the calyceal mucosa [61, 62]. Topical agents applied to the external parenchymal surface possess no sealing properties capable of withstanding the intraluminal hydrostatic pressure of the urinary collecting system.

To our knowledge, only one prior systematic review and meta-analysis has addressed a related question: Guo et al. (2021) [63] evaluated hemostatic agents versus standard suturing in PN and reported significant reductions in warm ischemia time, operative time, and EBL favoring hemostatic agents, without significant differences in BTR, HC, or UL. However, a fundamental methodological distinction separates that analysis from ours: Guo et al. evaluated hemostatic agents used as a sutureless alternative to renorrhaphy—a scenario where the observed benefits in operative time and ischemia time reflect the elimination of the suturing step itself rather than any pharmacological property of the agents. When renorrhaphy is retained and agents are added adjunctively—as in our study and in real-world practice—these benefits disappear entirely. The two reviews therefore address related but distinct questions and are complementary rather than competing, differing in scope (six studies there, fifteen here) and in analytic approach to heterogeneity. These differences in eligibility account for the divergent findings on operative and ischemia time and blood loss, and no inference about the relative merit of either review should be drawn from them.

The review has certain strengths. It is, to our knowledge, the most comprehensive synthesis of the adjunctive—as opposed to substitutive—use of these agents in PN, and BTR is a comparatively objective endpoint. Including both randomized and observational designs allowed efficacy and real-world effectiveness to be examined together, and the symmetric funnel plot with a non-significant Egger test provides some reassurance against selective reporting, though both tests have limited power with fifteen studies.

Several limitations constrain our conclusions. First, this is a null result from a low-certainty base with wide prediction intervals (BTR 0.24–3.90; HC 0.14–3.83; UL 0.45–4.09), each spanning important benefit and harm—an absence of evidence of benefit, not evidence of its absence. Second, the intervention is not a single entity: the pooled agents differ in composition and mechanism, and an agent-specific effect could be diluted to the null within a composite arm. Third, the literature spans two decades and three surgical eras with shifting, institution-defined transfusion thresholds, which could attenuate a real effect; we have removed our earlier claim that transfusion, as a hard endpoint, was robust to this. Fourth, all but two studies are non-randomized and none adjusted for indication, biasing the observational estimate toward the null. Fifth, EBL heterogeneity precluded pooling, and the meta-regressions were too underpowered to support inference. Finally, GRADE certainty was moderate only for the randomized transfusion evidence and low or very low elsewhere, so any clinical inference must remain provisional.

These data do not support routine, unselective use of hemostatic agents in PN, but neither do they establish that the agents are without effect. Reserving them for hemostatic rescue, coagulopathy, complex hilar resection, or the solitary kidney is reasonable, but this is a pragmatic clinical judgement, not a finding of this review: no included study evaluated a rescue role. The demonstrable conclusion concerns routine use in the unselected patient, where no benefit justifies the added cost—leaving selective use in high-risk settings open and in need of purpose-designed trials.

## Conclusions

This meta-analysis found no demonstrable benefit of adjunctive hemostatic agents over renorrhaphy alone for transfusion, hemorrhagic complications, or urinary leakage, while considerable heterogeneity precluded pooling for blood loss. With wide intervals and low-to-very-low certainty for most outcomes, these findings represent an absence of evidence of benefit rather than evidence of absence, and a clinically relevant effect cannot be excluded. Current data do not support routine use but cannot adjudicate selective use in high-risk settings, which adequately powered trials—stratified by tumor complexity and agent class—are needed to resolve.

## Supplementary Information

Below is the link to the electronic supplementary material.

Supplementary file1 (DOCX 823 KB)

## Data Availability

No datasets were generated or analysed during the current study.
