# Supplementary material for: Adjunctive topical hemostatic agents combined with renorrhaphy versus renorrhaphy alone during partial nephrectomy: a systematic review and meta-analysis
Source: World J Urol. 2026 Aug 21;44(1):592. doi: 10.1007/s00345-026-06679-5 (PMC13498511; doi:10.1007/s00345-026-06679-5)
Supplement: Supplementary file 1 [file 345_2026_6679_MOESM1_ESM.docx]

**Supplemental Online Content**

**eTable 1.** Search strategy

**eTable 2.** PRISMA 2020 checklist

**eTable 3.** Topical hemostatic agents employed in each included study

**eFigure 1.** Blood transfusion rate: leave-one-out analysis

**eFigure 2.** Blood transfusion rate: Baujat plot

**eFigure 3.** Estimated blood loss: forest plot (exploratory pooled estimate; presented for completeness only — see Methods)

**eFigure 4.** Estimated blood loss: leave-one-out analysis

**eFigure 5.** Estimated blood loss: Baujat plot

**eFigure 6.** Hemorrhagic complications: leave-one-out analysis

**eFigure 7.** Hemorrhagic complications: Baujat plot

**eFigure 8.** Blood transfusion rate: meta-regression by tumor size (exploratory)

**eFigure 9.** Hemorrhagic complications: meta-regression by tumor size (exploratory)

**eFigure 10.** Estimated blood loss: meta-regression by tumor size (exploratory)

**eFigure 11.** Blood transfusion rate: meta-regression by RENAL nephrometry score (exploratory)

**eFigure 12.** Hemorrhagic complications: meta-regression by RENAL nephrometry score (exploratory)

**eFigure 13.** Estimated blood loss: meta-regression by RENAL nephrometry score (exploratory)

**eTable 1.** Search Strategy

| PubMed (MEDLINE) | (("Nephrectomy"[MeSH Terms] OR "nephrectomy"[tiab] OR "partial nephrectomy"[tiab] OR "nephron sparing"[tiab] OR "nephron-sparing"[tiab] OR "kidney sparing"[tiab] OR "renorrhaphy"[tiab] OR "nephron preservation"[tiab])  AND  ("Hemostatics"[MeSH Terms] OR "hemostatic agent"[tiab] OR "hemostatic agents"[tiab] OR "haemostatic agent"[tiab] OR "haemostatic agents"[tiab] OR "FloSeal"[tiab] OR "Floseal"[tiab] OR "TachoSil"[tiab] OR "Tachosil"[tiab] OR "BioGlue"[tiab] OR "Bioglue"[tiab] OR "Surgiflo"[tiab] OR "Tisseel"[tiab] OR "Tissucol"[tiab] OR "fibrin glue"[tiab] OR "fibrin sealant"[tiab] OR "fibrin sealants"[tiab] OR "tissue adhesive"[tiab] OR "tissue adhesives"[tiab] OR "tissue sealant"[tiab] OR "tissue sealants"[tiab] OR "gelatin matrix"[tiab] OR "gelatin-thrombin"[tiab] OR "thrombin sealant"[tiab] OR "oxidized cellulose"[tiab] OR "Surgicel"[tiab] OR "Nu-Knit"[tiab] OR "NuKnit"[tiab] OR "Hemopatch"[tiab] OR "Veriset"[tiab] OR "hemostatic patch"[tiab] OR "haemostatic patch"[tiab] OR "hemostatic matrix"[tiab] OR "Evicel"[tiab] OR "Arista"[tiab] OR "CoSeal"[tiab] OR "Progel"[tiab] OR "Fibrin Tissue Adhesive"[MeSH Terms] OR "Tissue Adhesives"[MeSH Terms])) |
| --- | --- |
| Embase | (  ('kidney tumor'/exp OR 'renal tumor':ti,ab OR 'renal mass':ti,ab OR 'renal cell carcinoma':ti,ab)  AND  ('partial nephrectomy'/exp OR 'partial nephrectomy':ti,ab OR 'nephron sparing':ti,ab OR nephron-sparing:ti,ab OR 'kidney sparing':ti,ab OR renorrhaphy:ti,ab)  )  AND  (  ('hemostatic agent'/exp OR 'fibrin glue'/exp OR 'tissue adhesive'/exp  OR 'hemostatic agent*':ti,ab OR 'haemostatic agent*':ti,ab  OR 'fibrin sealant*':ti,ab OR 'tissue sealant*':ti,ab  OR floseal:ti,ab OR tachosil:ti,ab OR surgicel:ti,ab OR surgiflo:ti,ab  OR tisseel:ti,ab OR tissucol:ti,ab OR hemopatch:ti,ab OR veriset:ti,ab  OR 'oxidized cellulose':ti,ab OR 'gelatin matrix':ti,ab OR 'gelatin thrombin':ti,ab  )  ) |
| Cochrane (CENTRAL) | (([mh Nephrectomy] OR nephrectomy:ti,ab OR "partial nephrectomy":ti,ab OR "nephron sparing":ti,ab OR nephron-sparing:ti,ab OR "kidney sparing":ti,ab OR renorrhaphy:ti,ab OR "nephron preservation":ti,ab)  AND  ([mh Hemostatics] OR "hemostatic agent":ti,ab OR "hemostatic agents":ti,ab OR "haemostatic agent":ti,ab OR "haemostatic agents":ti,ab OR FloSeal:ti,ab OR Floseal:ti,ab OR TachoSil:ti,ab OR Tachosil:ti,ab OR BioGlue:ti,ab OR Bioglue:ti,ab OR Surgiflo:ti,ab OR Tisseel:ti,ab OR Tissucol:ti,ab OR "fibrin glue":ti,ab OR "fibrin sealant":ti,ab OR "fibrin sealants":ti,ab OR "tissue adhesive":ti,ab OR "tissue adhesives":ti,ab OR "tissue sealant":ti,ab OR "tissue sealants":ti,ab OR "gelatin matrix":ti,ab OR gelatin-thrombin:ti,ab OR "thrombin sealant":ti,ab OR "oxidized cellulose":ti,ab OR Surgicel:ti,ab OR Nu-Knit:ti,ab OR NuKnit:ti,ab OR Hemopatch:ti,ab OR Veriset:ti,ab OR "hemostatic patch":ti,ab OR "haemostatic patch":ti,ab OR "hemostatic matrix":ti,ab OR Evicel:ti,ab OR Arista:ti,ab OR CoSeal:ti,ab OR Progel:ti,ab OR [mh "Fibrin Tissue Adhesive"] OR [mh "Tissue Adhesives"])) |
| Web of Science | ((Nephrectomy OR nephrectomy OR "partial nephrectomy" OR "nephron sparing" OR nephron-sparing OR "kidney sparing" OR renorrhaphy OR "nephron preservation")  AND  (Hemostatics OR "hemostatic agent" OR "hemostatic agents" OR "haemostatic agent" OR "haemostatic agents" OR FloSeal OR Floseal OR TachoSil OR Tachosil OR BioGlue OR Bioglue OR Surgiflo OR Tisseel OR Tissucol OR "fibrin glue" OR "fibrin sealant" OR "fibrin sealants" OR "tissue adhesive" OR "tissue adhesives" OR "tissue sealant" OR "tissue sealants" OR "gelatin matrix" OR gelatin-thrombin OR "thrombin sealant" OR "oxidized cellulose" OR Surgicel OR Nu-Knit OR NuKnit OR Hemopatch OR Veriset OR "hemostatic patch" OR "haemostatic patch" OR "hemostatic matrix" OR Evicel OR Arista OR CoSeal OR Progel OR "Fibrin Tissue Adhesive" OR "Tissue Adhesives")) |
| Scopus | ((Nephrectomy OR nephrectomy OR "partial nephrectomy" OR "nephron sparing" OR nephron-sparing OR "kidney sparing" OR renorrhaphy OR "nephron preservation")  AND  (Hemostatics OR "hemostatic agent" OR "hemostatic agents" OR "haemostatic agent" OR "haemostatic agents" OR FloSeal OR Floseal OR TachoSil OR Tachosil OR BioGlue OR Bioglue OR Surgiflo OR Tisseel OR Tissucol OR "fibrin glue" OR "fibrin sealant" OR "fibrin sealants" OR "tissue adhesive" OR "tissue adhesives" OR "tissue sealant" OR "tissue sealants" OR "gelatin matrix" OR gelatin-thrombin OR "thrombin sealant" OR "oxidized cellulose" OR Surgicel OR Nu-Knit OR NuKnit OR Hemopatch OR Veriset OR "hemostatic patch" OR "haemostatic patch" OR "hemostatic matrix" OR Evicel OR Arista OR CoSeal OR Progel OR "Fibrin Tissue Adhesive" OR "Tissue Adhesives")) |

**eTable 2.** PRISMA 2020 checklist

| **Section and Topic** | **Item #** | **Checklist item** | **Location where item is reported** |
| --- | --- | --- | --- |
| **TITLE** |  |  |  |
| Title | 1 | Identify the report as a systematic review. | Title page ("A Systematic Review and Meta-Analysis") |
| **ABSTRACT** |  |  |  |
| Abstract | 2 | See the PRISMA 2020 for Abstracts checklist. | Abstract |
| **INTRODUCTION** |  |  |  |
| Rationale | 3 | Describe the rationale for the review in the context of existing knowledge. | Introduction (paras 1–5) |
| Objectives | 4 | Provide an explicit statement of the objective(s) or question(s) the review addresses. | Introduction (final para); Abstract (Purpose) |
| **METHODS** |  |  |  |
| Eligibility criteria | 5 | Specify the inclusion and exclusion criteria for the review and how studies were grouped for the syntheses. | Methods — Eligibility Criteria |
| Information sources | 6 | Specify all databases, registers, websites, organisations, reference lists and other sources searched or consulted to identify studies. Specify the date when each source was last searched or consulted. | Methods — Search Strategy (MEDLINE, Embase, CENTRAL, Web of Science, Scopus; reference lists; final search 15 March 2026) |
| Search strategy | 7 | Present the full search strategies for all databases, registers and websites, including any filters and limits used. | Methods — Search Strategy; eTable 1 |
| Selection process | 8 | Specify the methods used to decide whether a study met the inclusion criteria of the review, including how many reviewers screened each record and each report retrieved, whether they worked independently, and if applicable, details of automation tools used in the process. | Methods — Study Selection (two independent reviewers, third-party adjudication; no automation tools) |
| Data collection process | 9 | Specify the methods used to collect data from reports, including how many reviewers collected data from each report, whether they worked independently, any processes for obtaining or confirming data from study investigators, and if applicable, details of automation tools used in the process. | Methods — Data Extraction and Quality Assessment (two independent reviewers, standardized form, third reviewer for discrepancies) |
| Data items | 10a | List and define all outcomes for which data were sought. Specify whether all results that were compatible with each outcome domain in each study were sought, and if not, the methods used to decide which results to collect. | Methods — Outcomes (BTR primary; EBL, HC, UL secondary) |
|  | 10b | List and define all other variables for which data were sought. Describe any assumptions made about any missing or unclear information. | Methods — Data Extraction and Quality Assessment (study design, sample size, demographics, surgical approach, agent type) |
| Study risk of bias assessment | 11 | Specify the methods used to assess risk of bias in the included studies, including details of the tool(s) used, how many reviewers assessed each study and whether they worked independently, and if applicable, details of automation tools used in the process. | Methods — Data Extraction and Quality Assessment (RoB 2 for RCTs; ROBINS-I V2 for observational; two independent reviewers, structured consensus) |
| Effect measures | 12 | Specify for each outcome the effect measure(s) used in the synthesis or presentation of results. | Methods — Statistical Analysis (RR for dichotomous; MD for continuous) |
| Synthesis methods | 13a | Describe the processes used to decide which studies were eligible for each synthesis. | Methods — Eligibility Criteria; Statistical Analysis |
|  | 13b | Describe any methods required to prepare the data for presentation or synthesis, such as handling of missing summary statistics, or data conversions. | Methods — Statistical Analysis (proportional division of shared control groups per Cochrane Handbook to avoid unit-of-analysis errors) |
|  | 13c | Describe any methods used to tabulate or visually display results of individual studies and syntheses. | Methods — Statistical Analysis (forest plots; prediction intervals); Results (Figures 2–4) |
|  | 13d | Describe any methods used to synthesize results and provide a rationale for the choice(s). If meta-analysis was performed, describe the model(s), method(s) to identify the presence and extent of statistical heterogeneity, and software package(s) used. | Methods — Statistical Analysis (random-effects REML; I² with graded ranges and 95% confidence intervals, τ², Cochran's Q, 95% prediction intervals; R v4.0.0 / RStudio) |
|  | 13e | Describe any methods used to explore possible causes of heterogeneity among study results. | Methods — Statistical Analysis (meta-regression by tumor size and RENAL score; subgroup analyses by design and approach) |
|  | 13f | Describe any sensitivity analyses conducted to assess robustness of the synthesized results. | Methods — Statistical Analysis (leave-one-out and Baujat analyses) |
| Reporting bias assessment | 14 | Describe any methods used to assess risk of bias due to missing results in a synthesis (arising from reporting biases). | Methods — Statistical Analysis / Results (funnel plot and Egger test) |
| Certainty assessment | 15 | Describe any methods used to assess certainty (or confidence) in the body of evidence for an outcome. | Methods — Data Extraction and Quality Assessment (GRADE) |
| **RESULTS** |  |  |  |
| Study selection | 16a | Describe the results of the search and selection process, from the number of records identified in the search to the number of studies included in the review, ideally using a flow diagram. | Results — Study Selection and Characteristics (2,281 identified → 15 included); Figure 1 (PRISMA flow diagram) |
|  | 16b | Cite studies that might appear to meet the inclusion criteria, but which were excluded, and explain why they were excluded. | Figure 1 (PRISMA flow diagram, exclusion reasons) |
| Study characteristics | 17 | Cite each included study and present its characteristics. | Results — Study Selection and Characteristics; Table 1; eTable 3 |
| Risk of bias in studies | 18 | Present assessments of risk of bias for each included study. | Results — Risk of Bias Assessment; Figure 5 |
| Results of individual studies | 19 | For all outcomes, present, for each study: (a) summary statistics for each group and (b) an effect estimate and its precision, ideally using structured tables or plots. | Results — Meta-analysis; Figures 2–4; eFigure 3 |
| Results of syntheses | 20a | For each synthesis, briefly summarise the characteristics and risk of bias among contributing studies. | Results — Meta-analysis; Risk of Bias Assessment |
|  | 20b | Present results of all statistical syntheses conducted. If meta-analysis was done, present for each the summary estimate and its precision and measures of statistical heterogeneity. If comparing groups, describe the direction of the effect. | Results — Primary endpoint and Secondary outcomes (RR/MD, 95% CI, I², τ², prediction intervals) |
|  | 20c | Present results of all investigations of possible causes of heterogeneity among study results. | Results — Meta-regression; subgroup analyses; eFigures 8–13 |
|  | 20d | Present results of all sensitivity analyses conducted to assess the robustness of the synthesized results. | Results — Primary endpoint and Secondary outcomes (LOO, Baujat); eFigures 1–2, 4–7 |
| Reporting biases | 21 | Present assessments of risk of bias due to missing results (arising from reporting biases) for each synthesis assessed. | Results — Primary endpoint (Egger p = 0.598; funnel plot); Figure 3 |
| Certainty of evidence | 22 | Present assessments of certainty (or confidence) in the body of evidence for each outcome assessed. | Results — GRADE Assessment; Supplementary Material (GRADE evidence profile) |
| **DISCUSSION** |  |  |  |
| Discussion | 23a | Provide a general interpretation of the results in the context of other evidence. | Discussion (paras 1–7, incl. comparison with Guo et al.) |
|  | 23b | Discuss any limitations of the evidence included in the review. | Discussion — Limitations (agent heterogeneity, era/threshold drift, confounding by indication, certainty) |
|  | 23c | Discuss any limitations of the review processes used. | Discussion — Strengths and Limitations (limited power of Egger test; meta-regression fragility) |
|  | 23d | Discuss implications of the results for practice, policy, and future research. | Discussion (final two paras); Conclusions |
| **OTHER INFORMATION** |  |  |  |
| Registration and protocol | 24a | Provide registration information for the review, including register name and registration number, or state that the review was not registered. | Methods — Search Strategy (PROSPERO CRD420261396802) |
|  | 24b | Indicate where the review protocol can be accessed, or state that a protocol was not prepared. | Methods — Search Strategy (PROSPERO registration) |
|  | 24c | Describe and explain any amendments to information provided at registration or in the protocol. | Methods — Statistical Analysis (post hoc/exploratory nature of meta-regressions declared) |
| Support | 25 | Describe sources of financial or non-financial support for the review, and the role of the funders or sponsors in the review. | Declarations — Funding (Not applicable) |
| Competing interests | 26 | Declare any competing interests of review authors. | Declarations — Conflict of interest (Nothing to declare) |
| Availability of data, code and other materials | 27 | Report which of the following are publicly available and where they can be found: template data collection forms; data extracted from included studies; data used for all analyses; analytic code; any other materials used in the review. | Declarations — Data availability statement (available upon request) |

**eTable 3.** Topical hemostatic agents employed in each included study

| **Study** | **Year** | **Design** | **Approach** | **Hemostatic agent(s)** | **Agent class** |
| --- | --- | --- | --- | --- | --- |
| Gill | 2005 | Observational | Laparoscopic | FloSeal | Gelatin–thrombin matrix |
| Porpiglia | 2007 | Observational | Laparoscopic | Tissucol (fibrin glue) + collagen fleece | Fibrin sealant + collagen fleece |
| Siemer | 2007 | RCT | Open | TachoSil | Fibrin-coated collagen patch |
| Arnoux | 2013 | Observational | Open + laparoscopic | FloSeal (majority); also BioGlue, Quixil, TachoSil | Predominantly gelatin–thrombin matrix (mixed) |
| Cohen | 2013 | Observational | Robotic | Evicel | Fibrin sealant |
| Antonelli | 2015 | Observational | Open + minimally invasive | FloSeal vs TachoSil (two arms) | Gelatin–thrombin matrix; fibrin-coated collagen patch |
| Peyronnet | 2015 | Observational | Robotic | FloSeal, TachoSil, Surgicel, combinations | Mixed (gelatin–thrombin matrix / patch / oxidized cellulose) |
| Abu-Ghanem | 2016 | Observational | Open + laparoscopic | Cryoprecipitate–thrombin; BioGlue (± Surgicel) | Fibrin sealant / albumin-glutaraldehyde (mixed) |
| Maurice | 2016 | Observational | Robotic | Oxidized cellulose, fibrin sealants, gelatin-based, combinations | Mixed |
| Ozgor | 2016 | Observational | Laparoscopic | FloSeal | Gelatin–thrombin matrix |
| Carrion | 2017 | Observational | Laparoscopic | FloSeal + Surgicel | Gelatin–thrombin matrix + oxidized cellulose |
| Tonyali | 2017 | Observational | Laparoscopic | TachoSil vs FloSeal (two arms) | Fibrin-coated collagen patch; gelatin–thrombin matrix |
| Shigeta | 2020 | Observational | Laparoscopic | TachoSil | Fibrin-coated collagen patch |
| Staehler | 2022 | Observational | Open | Hemopatch | Polyethylene-glycol-coated collagen patch |
| Lazarovich | 2026 | RCT | Robotic | FloSeal / Surgiflo (± Nu-Knit) | Gelatin–thrombin matrix (± oxidized cellulose) |

Several studies compared or pooled more than one agent within a single intervention arm (Antonelli, Tonyali, Peyronnet, Maurice, Abu-Ghanem, Carrion, Arnoux). A pre-planned subgroup analysis by agent class was therefore not feasible. Agent classes differ substantially in composition and mechanism of action - gelatin–thrombin matrices act by mechanical tamponade with local thrombin-mediated clot formation, fibrin sealants by reconstituting the terminal coagulation cascade, collagen-based patches by contact activation and platelet aggregation, and oxidized cellulose by contact activation and a low-pH effect - and pooling them presupposes a class effect that has not been demonstrated.

**eFigure 1:** BTR Leave-one-out analysis

**eFigure 2:** BTR Baujat plot

**eFigure 3.** Estimated blood loss: forest plot (exploratory pooled estimate; presented for completeness only — see Methods)

Random-effects meta-analysis (REML) of estimated blood loss (mL), hemostatic agents plus renorrhaphy versus renorrhaphy alone. Negative values favour hemostatic agents. Heterogeneity was considerable (I² = 97.8%; τ² = 3,385.80; Q = 324.98, df = 7, p < 0.0001), and the 95% prediction interval extended from −162.11 to +131.56 mL. This pooled estimate is presented for completeness only and is not advanced as a finding of this review. As detailed in the Methods and Discussion, a single summary effect is not considered interpretable for this outcome, and estimated blood loss is reported narratively in the main text. Peyronnet (2015) contributed 84.6% of the common-effect weight.

**eFigure 4:** EBL Leave-one-out analysis

**eFigure 5:** EBL Baujat plot

**eFigure 6:** HC Leave-one-out analysis

**eFigure 7:** HC Baujat plot

**eFigures 8–13. Exploratory meta-regressions.**

Random-effects meta-regressions (REML) of tumor size and RENAL nephrometry score as candidate moderators of bleeding-related outcomes. Models were fitted on between three and ten studies. **None of these models satisfies the approximately ten-studies-per-covariate criterion recommended in the Cochrane Handbook, and the Handbook advises against meta-regression when fewer than ten studies are available.** All estimates are consequently subject to severe overfitting, are accompanied by confidence intervals spanning one to two orders of magnitude, and must be regarded as fragile. In particular, the model for RENAL score and blood transfusion rate (eFigure 11; k = 5) yields an implausibly large point estimate and an R² of 100%, a pattern characteristic of a model with minimal residual degrees of freedom rather than of a genuine dose–response relationship; neither its nominal statistical significance nor its apparent complete explanation of between-study variance should be interpreted literally. These analyses are presented as hypothesis-generating only and support no conclusion of this review.

**eFigure 8:** Blood transfusion rate: meta-regression by tumor size (exploratory)

**eFigure 9:** Hemorrhagic complications: meta-regression by tumor size (exploratory)

**eFigure 10:** Estimated blood loss: meta-regression by tumor size (exploratory)

**eFigure 11:** Blood transfusion rate: meta-regression by RENAL nephrometry score (exploratory)

**eFigure 12:** Hemorrhagic complications: meta-regression by RENAL nephrometry score (exploratory)

**eFigure 13:** Estimated blood loss: meta-regression by RENAL nephrometry score (exploratory)
